# The Principal Forms of the Skeleton and of the Teeth

**Published:** 1854-12

**Authors:** 


ART. VII.—*The principal forms of the Skeleton and of the Teeth.* By Prof. R. OWEN, F. R. S., &c.; Author of Odontography; Lectures on Comparative Anatomy, &c. Philadelphia: Blanchard & Lea. 1854. pp. 360, 16 mo.

This little work is from the pen of one of the highest authorities in our profession. Among all the eminent names that give lustre to British Science, there is not one which carries with it greater weight than that of Prof. OWEN, who is distinguished alike by his profound investigations, the originality and soundness of his opinions, and the eloquence with which he expresses his thoughts on scientific subjects. As a cultivator of Comparative Anatomy, he has no superior at the present day. His work, of which the title is given above, though diminutive in size, is no elementary treatise on the Skeleton and Teeth, but a philosophical disquisition on Osteology, presenting the true principles of that science. It is therefore not a book to be read cursorily, but one to be studied, and to the student of Zoology it is one of great interest. Its details are curious and instructive, and illustrated as the text is by numerous wood-cuts the reader will not find it difficult to follow the author in his descriptions. We copy his concluding remarks on the skeleton:

"A retrospect of the varied forms and proportions of the skeletons of animals, whether modified for aquatic, ærial, or terrestrial life, will show that whilst they were perfectly and beautifully adapted to the sphere of life and exigencies of the species, they adhered with remarkable constancy to that general pattern or archetype which was first manifested on this planet, as Geology teaches, in the class of fishes, and which has not been departed from even in the most extremely modified skeleton of the last and highest form which Creative Wisdom has been pleased to place upon this earth.

"It is no mere transcendental dream, but true knowledge and legitimate fruit of inductive research, that clear insight into the essential nature of each element of the bony framework, which is acquired by tracing them step by step, as *e. g.* from the unbranched pectoral ray of the lepidosiren to the equally small and slender but bifid pectoral ray of the amphiume, thence to the similar but trifid ray of the proteus, and through the progressively superadded strustures and perfections of the limbs in higher reptiles and in mammals. If the special homology of each part of the diverging appendage and its supporting arch are recognizable from man to the fish, we cannot close the mind's eye to the evidences of that higher law of archetypal conformity on which the very power of tracing the lower and more special correspondences depend.

"Buffon has well remarked, in the Introduction to his great work on Natural History: 'It is only by comparing that we can judge, and our knowledge turns entirely on the relations that things bear to those which resemble them and to those which differ from them; so, if there were no animals, the nature of man would be far more incomprehensible than it is.'

"And if this be true, as to man's general nature and powers, it is equally so with regard to his anatomical structure.

"In the same spirit our philosophic poet felt that—

> "'Tis the sublime of man,
> Our noontide majesty, to know ourselves
> Part and proportions of a wondrous whole.—Coleridge.

"Vertebrated animals of progressively higher grades of structure have existed at successive periods of time on this planet, and they were constructed on a common plan with those that still exist.

"Some have concluded, therefore, that the characters of a species became modified in successive generations, and that it was transmuted into a higher species; a reptile, *e. g.* into a mammal; an ape into a negro. Let us consider, therefore, the import and value of the osteological differences between the gorilla—the highest of all apes—and man, in reference to this 'transmutation hypothesis.' The skeleton of an animal may be modified to a certain extent by the action of the muscles. By the development of the processes, ridges and crests, the anatomist judges of the muscular power of the individual to whom a skeleton under comparison has appertained. A very striking difference from the form of the human cranium results from the development of certain crests and ridges for the attachment of muscles, in the great apes; but none of the more important differences, on which the naturalist relies for the determination of the genus and species of the orangs and chimpanzees, have such an origin or dependent relation. The great superorbital ridge, *e. g.* againgt which the facial line rests in Fig. 50, is not the consequence of muscular action or development: it is characteristic of the genus *Troglodytes* from the time of birth; and we have no grounds for believing it to be a character to be gained or lost through the operations of external causes, inducing particular habits through successive generations of a species.

"No known cause of change productive of varieties of mammalian species could operate in altering the size, shape or connections of the prominent premaxillary bones, which so remarkably distinguish the great *Troglodytes gorilla* from the lowest races of mankind. There is not, in fact, any other character than that founded upon the development of bone for the attachment of muscles, which is known to be subject to change through the operation and influence of external causes. Nine-tenths of the differences which have been cited (see the *Transactions of the Zoological Society*, vol. iii. p. 413), as distinguishing the great chimpanzee from the human species, must stand in contravention of the hypothesis of transmutation and progressive development, until the acceptors of that hypothesis are enabled to adduce the facts demonstrative of the conditions of the modifiability of such characters. Moreover as the generic forms of the ape tribe approach the human type, they are represented by fewer species. The unity of the human species is demonstrated by the constancy of those osteological and dental peculiarities which are seen to be most characteristic of the *bimana* in contradistinction fron the *quadrumana*.

"Man is the sole species of his genus (*homo*)—the sole representative of his order (*bimana*); he has no nearer physical relations with the brute kind than those that belong to the characters which link together the unguiculate division of the mammalian class.

"Of the nature of the creative acts by which the successive races of animals were called into being, we are ignorant. But this we know, that as the evidence of unity of plan testifies to the oneness of the Creator, so the modifications of the plan for different modes of existence illustrate the beneficence of the Designer. Those structures, moreover, which are at present incomprehensible as adaptations to a special end, are made comprehensible on a higher principle, and a final purpose is gained in relation to human intelligence; for in the instances where the analogy of humanly invented machines fails to explain the structure of a divinely created organ, such organ does not exist in vain if its truer comprehension, in relation to the Divine idea, or prime Exemplar, lead rational beings to a better conception of their own origin and Creator."

